# Blood nutrition-related biomarkers for central nervous system injury rehabilitation prognosis: a retrospective study

**DOI:** 10.1186/s12944-026-02957-8

**Published:** 2026-04-28

**Authors:** Guiqing Cheng, Kun Zhao, Xinge Yu, Wenhui Ding, Huilin Liu, Fen Ju, Wei Sun, Fei Tian, Feng Feng, Xinyu Liu, Tao Han, Yixing Lu, Xiaolong Sun, Hua Yuan

**Affiliations:** 1 Department of Rehabilitation Medicine, XiJing Hospital, Air Force Medical University (The Fourth Military Medical University), Xi’an, Shaanxi China; 2 Department of Anesthesiology, The 945th Hospital of Joint Logistics Support Force of the Chinese People’s Liberation Army, Yaan, Sichuan China; 3 Department of Rehabilitation Medicine, The 940th Hospital of Joint Logistics Support Force of the Chinese People’s Liberation Army, Lanzhou, Gansu China

**Keywords:** Central nervous system, Spinal cord injury, Stroke, Prognosis, Biomarkers, Albumins, Cholesterol, Lipoproteins, LDL

## Abstract

**Background:**

Malnutrition is highly prevalent among patients with central nervous system (CNS) injuries. Although nutrition-related blood biomarkers help evaluate malnutrition, their prognostic value during CNS injury rehabilitation remains unclear.

**Objective:**

This study aimed to evaluate the impact of nutrition-related blood biomarkers on rehabilitation outcomes in CNS injury patients.

**Methods:**

A total of 669 patients with CNS injuries admitted to a Chinese tertiary teaching hospital from December 2017 to December 2025 were enrolled. Functional outcomes were assessed using the mean relative functional gain (mRFG) of the modified Barthel index. Independent prognostic factors were identified using both univariate and multivariate analyses, including demographic and clinical characteristics, complications, and blood biomarkers. The receiver operating characteristic (ROC) curve analysis was employed to assess the optimal cutoff values for independent predictors. Findings were validated using restricted cubic spline (RCS) models, subgroup analyses, and sensitivity analyses.

**Results:**

Multivariate analysis demonstrated that albumin (odds ratio [OR] = 0.934, *P* = 0.029), total cholesterol (TC, OR = 1.816, *P* < 0.001), and low-density lipoprotein cholesterol (LDL-C, OR = 1.580, *P =* 0.003) were independent predictors of functional prognosis. When grouped by cutoff values, significant differences in mRFG were observed for albumin (cutoff value = 36.35 g/L, *P* = 0.002), TC (cutoff value = 3.24 mmol/L, *P* < 0.001), and LDL-C (cutoff value = 1.89 mmol/L, *P* < 0.001). RCS analyses demonstrated no significant nonlinear relationships. Subgroup analyses revealed no significant effect modification by injury type, although TC showed significant positive associations in spinal cord injury and hemorrhagic stroke. A significant gender interaction was found for TC (*P* for interaction = 0.021), with a stronger association in males (OR = 2.43, 95% CI: 1.70–3.48).

**Conclusion:**

Albumin, TC, and LDL-C are independent predictors of functional prognosis in patients with CNS injury. Albumin is protective, while TC and LDL-C correlate with poorer outcomes. The identified cutoff values may help clinicians guide patient management and prognostic assessment.

**Supplementary Information:**

The online version contains supplementary material available at 10.1186/s12944-026-02957-8.

## Background

Central nervous system (CNS) injury encompassing spinal cord injuries (SCI) and brain injuries [1], significantly contribute to global mortality and disability [2], resulting in major economic pressures on healthcare systems and patients’ families. CNS injuries severely impair the ability to perform activities of daily living (ADL) [3], reduce mobility like walking and transferring, and adversely affect functional independence and quality of life, leading to muscle atrophy and lower food intake. Concurrently, CNS injuries often cause complications such as dysphagia and impaired feeding ability, which result in inadequate energy intake and an increased risk of malnutrition. The World Health Organization (WHO) charactrerizes malnutrition as an imbalances, deficiency, or excesses in a person’s energy or nutrients intake [4]. During the rehabilitation process, it is essential to ensure adequate nutritional support with sufficient energy, protein, fat, and other essential nutrients. This support helps mitigate lipid peroxidation and oxidative stress [5], thereby promoting recovery.

Despite the crucial role of nutrition in preventing complications and supporting neurological recovery, nutritional status is frequently overlooked in clinical practice. WHO has identified malnutrition as a significant global public health threat [6]. Studies indicate that 20%–60% of hospitalized patients experience malnutrition [7], which increases the risk of complications, prolongs length of stay (LOS), raises hospitalization costs, and ultimately elevates rehabilitation expenses. Therefore, early identification and management of nutrition-related abnormalities in patients with CNS injury are imperative.

Nutrition-related blood biomarkers, as part of routine admission blood tests, offer objective measures for assessing the degree of malnutrition and predicting rehabilitation outcomes. For instance, albumin—a widely studied protein—have been confirmed to associate with orthopedic postoperative complications and perioperative intraperitoneal chemotherapy outcome [8, 9]. Though the widespread use of blood biomarkers for nutritional assessment in hospitalized patients, their prognostic value for functional recovery during rehabilitation following CNS injury remains poorly defined. In light of this, the current study retrospectively investigated blood nutrition-related biomarkers influencing the rehabilitation prognosis of patients with CNS injury for public health strategies and clinical management.

## Methods

### Study population (participants)

This retrospective observational single-center study was conducted at a Chinese tertiary teaching hospital. The study protocol was approved by the Institutional Review Board of the medical center (SCI: KY20222096-C-1, brain injuries: KY20232243-C-1) and was registered in the Chinese Clinical Trial Registry (SCI: ChiCTR2300073824, brain injuries: ChiCTR2400081107). The study adhered to the principles of the Declaration of Helsinki, and informed consent was waived because of the retrospective study design.

The study involved hospitalized patients aged 18 years diagnosed of CNS injury from December 2017 to December 2025. CNS injuries primarily included SCI, hemorrhagic stroke, ischemic stroke, hypoxic–ischemic encephalopathy (HIE), intracranial tumors, and traumatic brain injury (TBI), confirmed by computed tomography (CT) or magnetic resonance imaging (MRI). Patients with missing core outcome data or any of the primary blood biomarkers were excluded. The exclusion criteria comprised: (1) LOS < 7 days; (2) thyroid diseases (hypothyroidism or hyperthyroidism); (3) severe systemic diseases, including cancer and psychiatric disorders; (4) other wasting diseases; (5) hematologic disorders such as leukemia, aplastic anemia; (6) prior immunomodulatory therapy (biologics, corticosteroids and methotrexate) or radiotherapy; (7) hepatic or renal insufficiency; (8) rehabilitation outcomes exhibiting ceiling effects [10], characterized by scores within the upper 5% of the maximum modified Barthel index (MBI) range.

### Data collection

Baseline characteristics, such as demographic data, comorbidities, injury profiles, and ADL assessments, were systematically collected for all enrolled patients.

Age and sex were recorded during hospital admission. Educational attainment was self-reported and classified into three levels: (1) Low (junior high school or below); (2) Medium (senior or vocational high school); (3) High (college or above) [11]. Smoking status was self-reported and was assessed as a dichotomous (yes or no) response. Non-smokers were individuals with no lifetime smoking history, while smokers included both former smokers (abstinent at CNS injury onset) and current smokers [12]. Diabetes, hypertension, and coronary heart disease (CHD) were confirmed based on medical histories or physician diagnosis. Pressure ulcers were assessed by nursing staff upon admission. Body mass index (BMI) was calculated utilizing the formula BMI = kg/m^2^ and categorized into four groups (underweight: BMI < 18.5 kg/m^2^, normal: 18.5 ≤ BMI < 25 kg/m^2^, overweight: 25 ≤ BMI < 30 kg/m^2^, obese: BMI ≥ 30 kg/m^2^) according to previous literature [13]. Time from lesion to rehabilitation facility (TLRF) was recorded as the days elapsed between injury onset and admission to inpatient rehabilitation [14]. Injury type (SCI, hemorrhagic stroke, ischemic stroke, intracranial tumor, HIE, or TBI) as well as feeding type (oral, enteral, or parenteral nutrition) were documented at admission. During hospitalization, nutritional support was limited to standard dietary provision as part of routine care, with no additional specialized interventions.

### Blood biomarker measurement

Blood biomarkers—including white blood cell (WBC), hemoglobin, total protein, albumin, creatinine, total cholesterol (TC), triglyceride (TG), high-density lipoprotein (HDL), and low-density lipoprotein cholesterol (LDL-C)—were analyzed as continuous variables. Following an 8-h fast, blood specimens were obtained within 24-h of admission by trained nurses and processed by the clinical laboratory based on standardized protocols.

### Outcome assessment

The MBI at admission and discharge was employed to assess the ADL of patients. MBI is a widely used tool in neurological rehabilitation and evaluates ten domains of functional independence associated with self-care and mobility [15]. Responses for each item are recored on a five-point scale (1: complete dependence; 5: complete independence), with a maximum total score of 100. Since the MBI does not inherently account for baseline injury severity or rehabilitation potential, the mean relative functional gain (mRFG) was employed to more accurately quantify functional recovery. The mRFG was calculated as follows: mRFG = (discharge MBI–admission MBI)/ (100–admission MBI) × 100% [16]. This formula adjusts for baseline functional status and mitigates the “ceiling effects”, in which patients with high admission MBI scores have limited potential for measurable improvement compared to those with lower initial scores [17]. mRFG > 0.5 was defined as good prognosis.

### Statistical analysis

The Kolmogorov–Smirnov test was used to assess the distribution of continuous variables. Varibles with a normal distribution were expressed as mean ± standard deviation (SD), while skewed variables were reported as median with interquartile range (IQR). Categorical variables were assessed using the chi-square test or Fisher’s exact test, while continuous variables with non-normal distributions were evaluated using the Mann-Whitney U test. For covariates with missing values (missing rate < 5%), single imputation was applied by replacing missing continuous values with the variable mean and missing categorical values with the mode.

To assess the association between blood nutrition biomarkers and rehabilitation outcomes, univariate and multivariate logistic regression models were utilized. Poor prognosis defined as an mRFG score ≤ 0.5, was treated as the event of interest (coded as 1) while good prognosis (mRFG > 0.5) constituted the reference category (coded as 0). Values of *P* < 0.05 in univariate analyses were selected for inclusion in multivariate logistic regression. An extended logistic model approach was applied to different covariate-adjusted models, resulting three models: (1) a crude model without any adjustments; (2) model 1 adjusted for gender, age, education, smoking, and BMI; (3) model 2 further adjusted for all other factors.

The restricted cubic spline (RCS) analyses were performed to flexibly model the association of albumin, TC, and LDL-C with prognosis; the presence of confusion was assessed by including or excluding covariates, in which albumin, TC, and LDL-C were mutually adjusted. The receiver operating characteristic (ROC) curve analysis was employed to assess the best cutoff value of albumin, TC, and LDL-C for discriminating between patients with good prognosis and poor prognosis. Subsequently, patients were categorized according to these cutoffs, and intergroup differences in prognosis were compared. Subgroup analyses grouped by injury type and gender were executed to determine the consistency of the association.

Multiple sensitivity analyses were executed to comprehensively assess the robustness of the primary findings: (1) Bootstrap resampling (1000 replicates) was applied to the multivariable logistic model to obtain more robust estimates; (2) the analysis was repeated using the continuous mRFG score in a multivariable linear regression model to verify that key associations were not dependent on outcome dichotomization; (3) potential multicollinearity was addressed by running separate models including either TC or LDL-C; (4) the influence of excluded cases was evaluated by re-including the 19 patients previously excluded due to the ceiling effect; and (5) a complete-case analysis was performed after excluding missing values to assess the potential impact of data missingness. All analyses utilized R Statistical Software (version 4.2.2; R Foundation for Statistical Computing, Vienna, Austria) and the Free Statistics analysis platform (version 2.0; Beijing, China), setting statistical significance at *P* < 0.05.

## Results

### Study participants

**Fig. 1:**
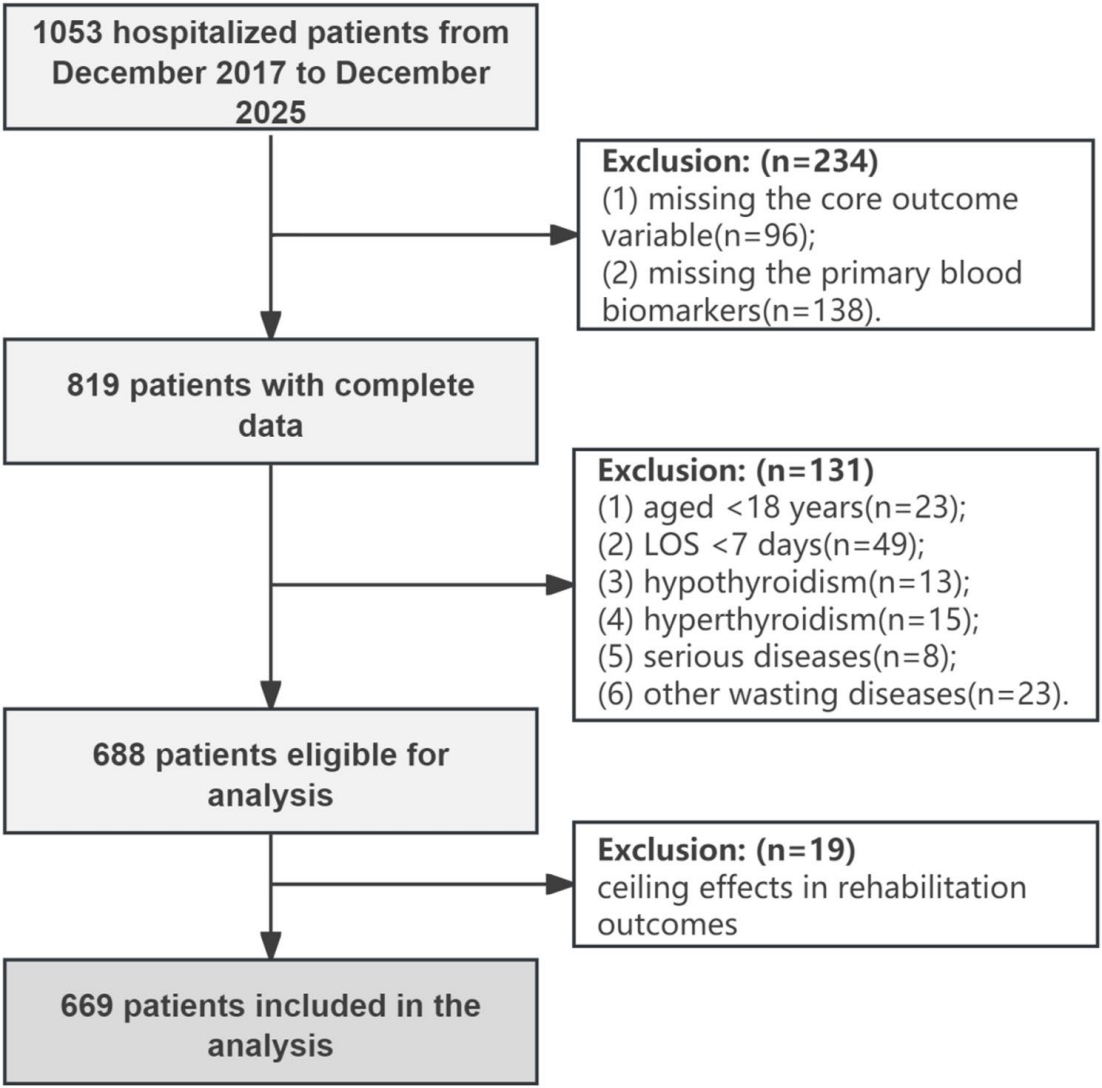
Flowchart of patients inclusion and exclusion. Abbreviations: LOS, length of stay

A total of 1053 hospitalized patients were initially screened from December 2017 to December 2025. After excluding 234 patients with incomplete data (the core outcome variable or any of the primary blood biomarkers missing) and 131 patients did not satisfy the inclusion criteria, 688 patients were deemed eligible for analysis. Subsequently, 19 additional patients were excluded due to ceiling effects in rehabilitation outcomes, resulting in a final cohort of 669 consecutive hospitalized patients (Fig. 1). Among these, 290 had SCI (43.35%), 166 had hemorrhagic stroke (24.81%), 161 had ischemic stroke (24.06%), 8 had intracranial tumors (1.20%), 2 had HIE (0.30%), and 42 had TBI (6.28%). The cohort included 503 males (75.19%) and 166 females (24.81%), with a median age of 52 (IQR : 41–63) years.

### Univariate analysis of baseline characteristics and nutrition-related blood biomarker with outcomes

Patients were stratified into a good prognosis group (*n* = 106, mRFG > 0.5) and a poor prognosis group (*n* = 563, mRFG ≤ 0.5). Table 1 illustrates the demographic and clinical characteristics, and complications of the two groups. There were no significant differences in demographic characteristics. In terms of clinical characteristics and complications, the TLRF in the poor prognosis group was significantly longer compared to that in the good prognosis group (Z = − 4.285, *P* < 0.001). Table 2 presents the comparison of blood biomarkers, demonstrated that patients with the good prognostic exhibit higher levels of hemoglobin (Z = − 2.376, *P* = 0.017), total protein (Z = − 2.420, *P* = 0.016), albumin (Z = − 3.119, *P* = 0.002), and creatinine (Z = − 2.337, *P* = 0.019) than those with the poor prognostic group at admission. Simultaneously, levels of TC (Z = − 4.279, *P* < 0.001), TG (Z = − 2.205, *P* = 0.027), and LDL-C (Z = − 3.495, *P* < 0.001) were observed notably lower in the good prognosis group compared to the poor prognosis group.

**Table 1:** Baseline admission characteristics and univariate analysis of factors associated with prognosis

Variables	Poor prognostic	Good prognostic	χ²/Z	*P*
	*n* = 563	*n* = 106		
Demographic characteristics				
Gender			0.102	0.750
Male	422	81		
Female	141	25		
Age, median (IQR), y	53 (41, 63)	51 (39, 60)	-1.025	0.305
Education			2.977	0.226
Low	94	19		
Medium	319	51		
High	150	36		
Smoking			0.749	0.397
Yes	107	24		
No	456	82		
BMI			3.816	0.282
Underweight	20	7		
Normal	381	63		
Overweight	147	33		
Obese	15	3		
Clinical characteristics				
TLRF, median (IQR), d	80 (40, 170)	35 (17, 104)	-4.285	**< 0.001**
Injury type			7.082	0.069
SCI	256	34		
Hemorrhagic stroke	137	29		
Ischemic stroke	128	33		
Other brain injury	42	10		
LOS, median (IQR), d	26 (14, 29)	26 (16, 33)	-1.025	0.305
Feeding type			0.003	0.988
Oral	404	76		
Enteral nutrition	154	29		
Parenteral nutrition	5	1		
Complications				
Diabetes			0.292	0.589
Yes	69	15		
No	494	91		
Hypertension			1.117	0.291
Yes	224	48		
No	339	58		
CHD			1.472	0.225
Yes	35	10		
No	528	96		
Pressure			1.820	0.177
Yes	27	2		
No	536	104		

underweight: BMI < 18.5 kg/m^2^, normal: 18.5 ≤ BMI < 25 kg/m^2^, overweight: 25 ≤ BMI < 30 kg/m^2^, obese: BMI ≥ 30 kg/m^2^

Significant values are in bold

*Abbreviations*: *BMI *body mass index, *TLRF *time from lesion to the current rehabilitation facility, *SCI *spinal cord injury, *LOS *length of stay, *CHD *coronary heart disease

**Table 2:** Blood biomarkers at admission and univariate analysis to determine the factors associated with prognosis

Variables	Poor prognostic	Good prognostic	t/Z	*P*
	*n* = 563	*n* = 106		
WBC, median (IQR), 10^9^/L	6.19 (5.09, 7.07)	6.52 (5.17, 8.40)	-0.881	0.378
Hemoglobin, median (IQR), g/L	129.00 (118.00, 141.00)	135.00 (124.75, 142.00)	-2.376	**0.017**
Total protein, median (IQR), g/L	64.20 (60.50, 67.50)	65.50 (61.80, 69.03)	-2.420	**0.016**
Albumin, median (IQR), g/L	37.90 (35.00, 40.10)	38.75 (36.98, 41.50)	-3.119	**0.002**
Creatinine (IQR), µmol/L	55.00 (46.00, 70.00)	59.00 (50.00, 73.00)	-2.337	**0.019**
TC, median (IQR), mmol/L	3.78 (3.24, 4.44)	3.35 (2.82, 4.08)	-4.279	**< 0.001**
TG, median (IQR), mmol/L	1.37 (1.01, 1.85)	1.26 (0.94, 1.64)	-2.205	**0.027**
HDL, median (IQR), mmol/L	0.92 (0.81, 1.05)	0.93 (0.80, 1.05)	-0.114	0.909
LDL-C, median (IQR), mmol/L	2.32 (1.83, 2.91)	1.93 (1.47, 2.71)	-3.495	**< 0.001**

Significant values are in bold

*Abbreviations*: *WBC *white blood cell, *TC *total cholesterol, *TG *triglyceride, *HDL *high-density lipoprotein, *LDL-C *low-density lipoprotein cholesterol

### Determination of nutrition-related blood biomarker affecting functional prognosis (multivariate analysis)

Variables with *P* < 0.05 in univariate analysis were included in multivariate models. Three models were constructed to control confounding factors (Table 3): a crude model without any adjustments; model 1 adjusted for gender, age, education, smoking, and BMI; and model 2 further adjusted for all other factors. The multivariate analysis indicated that albumin (odds ratio [OR] = 0.934, *P* = 0.029), TC (OR = 1.816, *P* < 0.001), and LDL-C (OR = 1.580, *P =* 0.003) remained significant after controlling for multiple potential confounders, confirming their status as independent influencing factors. Specifically, higher levels of albumin were related to a reduced risk of poor functional outcomes, whereas higher TC and LDL-C were linked to an higher risk of poor functional outcomes. Albumin, TC, and LDL-C emerged from multivariate analysis as robust independent predictors of functional outcomes, underscoring the clinical relevance of these nutritional biomarkers in the prognosis of CNS injury.

**Table 3:** Multivariate logistic regression of mRFG

Variables	Crude		Model 1		Model 2	
	OR (95% CI)	*P*	OR (95% CI)	*P*	OR (95% CI)	*P*
TLRF	1.001 (1.000 ~ 1.002)	0.062	1.001 (1.000 ~ 1.002)	0.056	1.001 (1.000 ~ 1.002)	0.062
Total protein	0.968 (0.932 ~ 1.005)	0.088	0.972 (0.935 ~ 1.010)	0.147	0.991 (0.951 ~ 1.032)	0.650
Hemoglobin	0.989 (0.977 ~ 1.000)	0.059	0.989 (0.976 ~ 1.002)	0.090	0.994 (0.981 ~ 1.008)	0.397
Albumin	0.915 (0.866 ~ 0.966)	**0.002**	0.918 (0.866 ~ 0.974)	**0.004**	0.934 (0.878 ~ 0.993)	**0.029**
Creatinine	0.998 (0.992 ~ 1.005)	0.615	0.998 (0.992 ~ 1.005)	0.591	1.000 (0.991 ~ 1.008)	0.946
TC	1.731 (1.352 ~ 2.218)	**< 0.001**	1.793 (1.384 ~ 2.324)	**< 0.001**	1.816 (1.368 ~ 2.411)	**< 0.001**
TG	1.040 (0.910 ~ 1.190)	0.564	1.051 (0.902 ~ 1.224)	0.524	1.033 (0.905 ~ 1.179)	0.631
LDL-C	1.644 (1.244 ~ 2.173)	**< 0.001**	1.688 (1.262 ~ 2.257)	**< 0.001**	1.580 (1.165 ~ 2.144)	**0.003**

Crude model without any adjustments; Model 1 adjusted for gender, age, education, smoking, BMI; Model 2 adjusted for all other factors

Significant values are in bold

*Abbreviations*: *CI *confidence interval, *TLRF *time from lesion to the current rehabilitation facility, *TC *total cholesterol, *TG *triglyceride, *LDL-C *low-density lipoprotein cholesterol

### Nonlinear relationships between albumin, TC, LDL-C, and functional prognosis

**Fig. 2:**
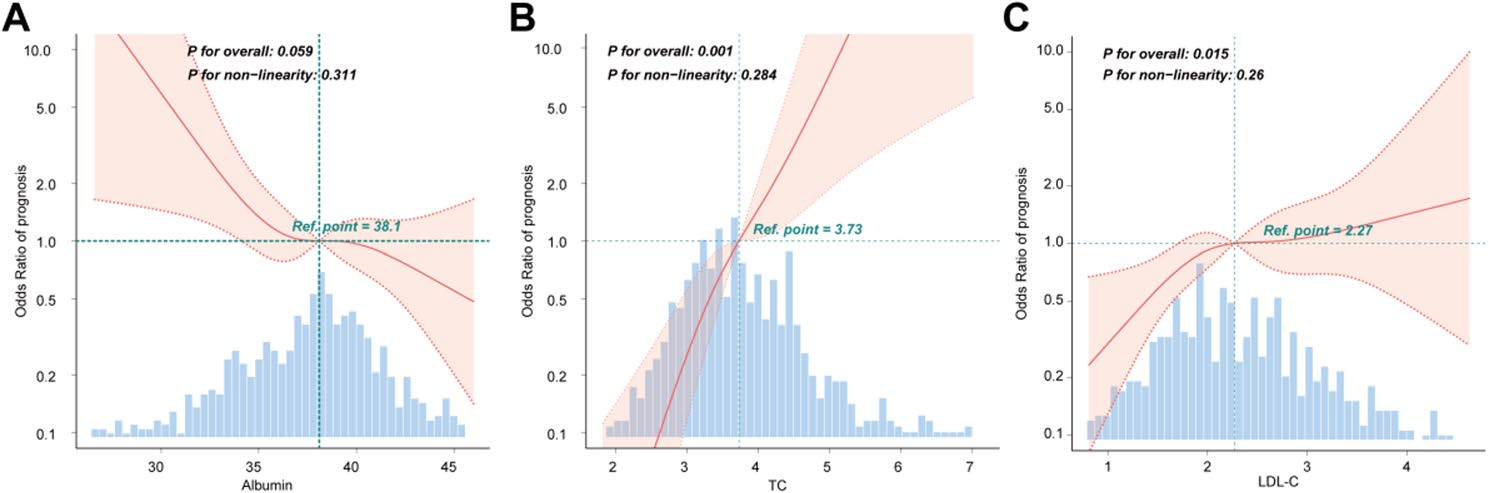
Testing for nonlinearity of albumin, TC, and LDL-C in CNS injury prognosis. **A** Testing for nonlinearity of albumin and mRFG. **B** Testing for nonlinearity of TC and mRFG. **C** Testing for nonlinearity of LDL-C and mRFG. Adjusted for all other factors. Albumin, TC, and LDL-C were also mutually adjusted. Solid lines depict predicted values; dashed lines represent 95% CI. Only 98% of the data are shown. Abbreviations: TC, total cholesterol; LDL-C, low-density lipoprotein cholesterol; CI, confidence interval

RCS analyses were performed to flexibly model the relationship between albumin, TC, LDL-C, and functional prognosis, with albumin, TC, and LDL-C being mutually adjusted. To ensure robustness, extreme values (top/bottom 0.5%) were excluded. Non-significant nonlinear associations were identified (Fig. 2). Trend analyses demonstrated reducing risks of poor prognosis with higher albumin levels, whereas elevated TC and LDL-C levels correlated with higher risks of unfavorable outcomes.

### Assessment of albumin, TC, and LDL-C cutoff values in mRFG

**Fig. 3:**
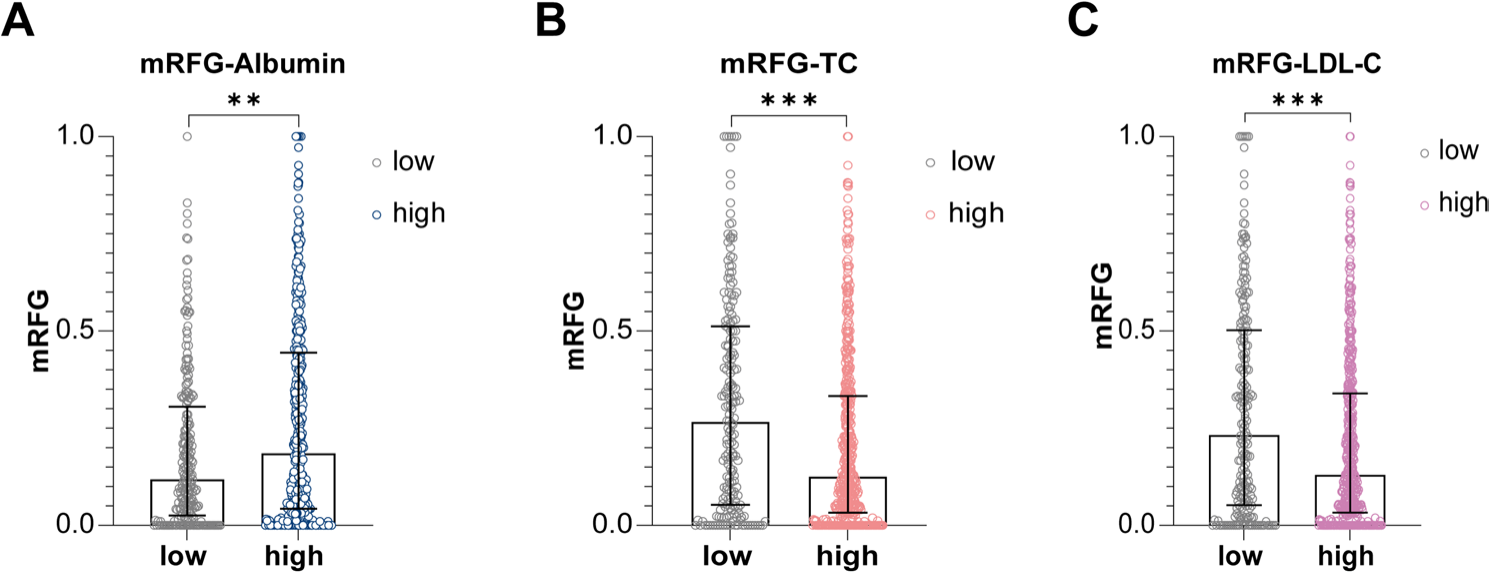
Assessment of albumin, TC, and LDL-C cutoff values in mRFG. **A** Assessment of albumin cutoff values in mRFG. **B** Assessment of TC cutoff values in mRFG. **C** Assessment of LDL-C cutoff values in mRFG. Abbreviations: mRFG, the mean relative functional gain; TC, total cholesterol; LDL-C, low-density lipoprotein cholesterol

ROC analysis was performed to determine the optimal cutoff values of albumin, TC, and LDL-C for predicting mRFG. The optimal cutoff values were 36.35 g/L for albumin, 3.24 mmol/L for TC, and 1.89 mmol/L for LDL-C, with corresponding area under the curve (AUC) values of 0.60 (95% confidence interval [CI]: 0.54–0.65), 0.63 (95% CI: 0.57–0.69), and 0.61 (95% CI: 0.55–0.67), respectively. Furthermore, albumin, TC, and LDL-C were grouped by these cutoff values, and the differences in prognosis between groups were compared (Fig. 3). There were significant differences in mRFG among groups stratified by albumin (*P =* 0.002), TC (*P* < 0.001) and LDL-C (*P* < 0.001).

### Stratified analyses and sensitivity analysis

**Fig. 4:**
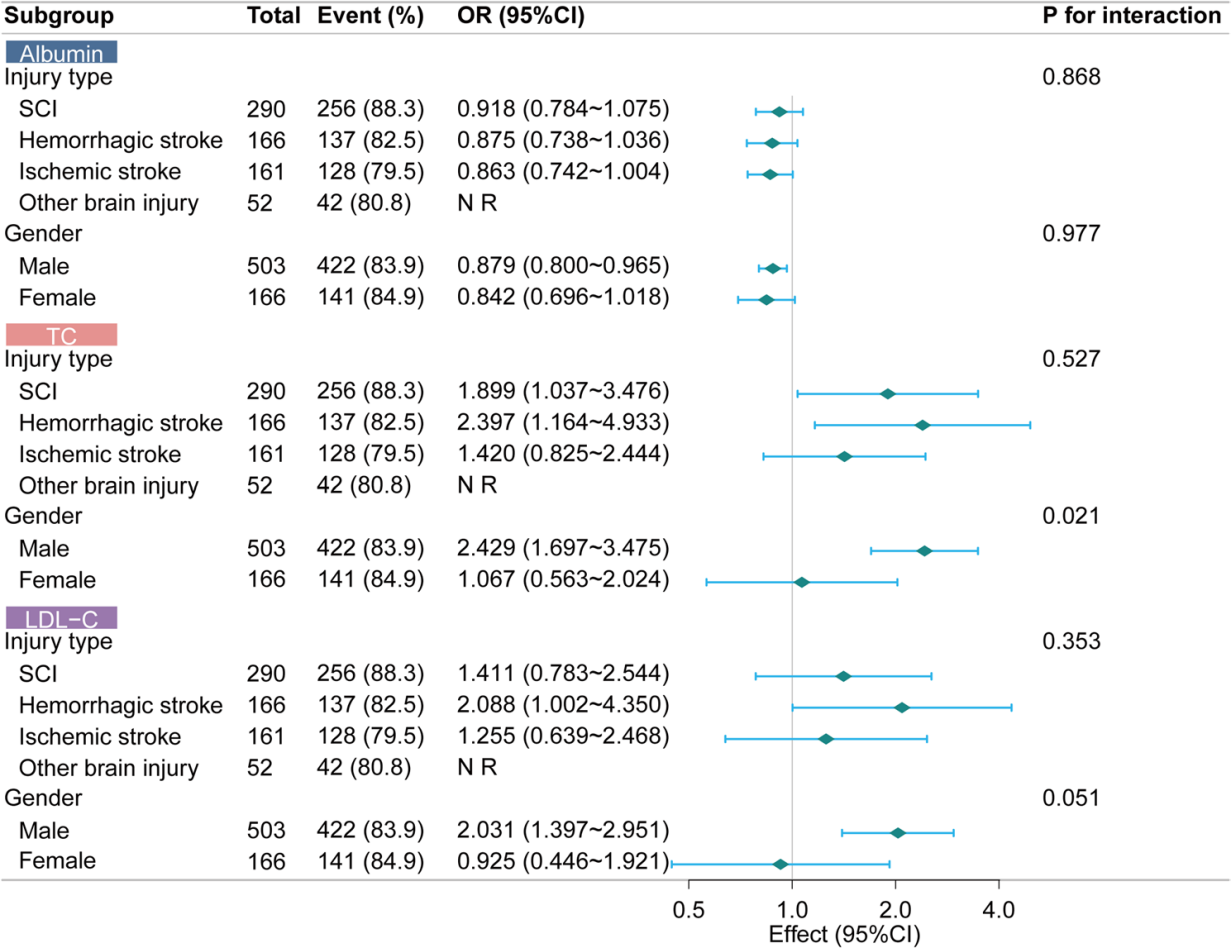
Forest plots for the association of albumin, TC, and LDL-C with prognosis. Adjusted for age, education, LOS, BMI, smoking, diabetes, hypertension, CHD, pressure, TLRF, feeding type, WBC, hemoglobin, total protein, creatinine, TG, and HDL. Abbreviations: CI, confidence interval; SCI, spinal cord injury; NR, non-reportable TC, total cholesterol; LDL-C, low-density lipoprotein cholesterol

Subgroup analyses were undertaken to evaluate the consistency of the associations of albumin, TC, and LDL-C with mRFG across injury types and between genders (Fig. 4). Due to limited sample size (*n* = 52), reliable effect estimates (OR and 95% CI) could not be computed for the “Other brain injury” subgroup, leading to non-reportable (NR) effect estimates in the result table. Among the remaining three injury types (SCI, hemorrhagic stroke, and ischemic stroke), no significant effect modification was observed for any of the three biomarkers (*P* for interaction = 0.868, 0.527, and 0.353, respectively), indicating no statistically significant heterogeneity across these three injury types. For albumin, the association with the outcome was not significant in any of the three injury subgroup (all ORs close to 1, 95% CI included 1), suggesting a consistent, non-significant protective trend. For TC, although the interaction was not significant, a distinct pattern was observed: a significant positive association was found in SCI (OR = 1.899, 95% CI: 1.037–3.476) and hemorrhagic stroke (OR = 2.397, 95% CI: 1.164–4.933), while positive but non-significant trends were seen in ischemic stroke (OR = 1.420, 95% CI: 0.825–2.444). For LDL-C, no significant association was found among the remaining three injury types (*P* for interaction = 0.353), while a significant positive association was found in hemorrhagic stroke (OR = 2.088, 95% CI: 1.002–4.350). In summary, injury type did not significantly modify the biomarker-outcome relationships overall. In gender subgroup analyses, a statistically significant interaction was found for TC (*P* for interaction = 0.021), suggesting a stronger association in male patients (OR = 2.429, 95% CI: 1.697–3.475), while the interaction for LDL-C approached significance (*P* for interaction = 0.051). No significant interaction with gender was found for albumin (*P* for interaction = 0.977), while the association with functional prognosis was significant in male patients for both TC (OR = 2.429, 95% CI: 1.697–3.475) and LDL-C (OR = 2.031, 95% CI: 1.397–2.951), but not in females. To evaluate the strength of the main results, a series of sensitivity analyses were conducted (Supplementary Tables 1–7 and Supplementary Figs. 1–2), including the re-inclusion of 19 patients previously excluded due to ceiling effects, Bootstrap resampling (1000 iterations), separate inclusion of TC and LDL-C to evaluate potential collinearity, linear regression using the continuous mRFG score, and a complete-case analysis after excluding missing data. All sensitivity analyses remained consistent with the core conclusions of the primary multivariable logistic regression model, further supporting the robustness of serum albumin, TC, and LDL-C as independent factors associated with functional prognosis.

## Discussion

CNS injuries are critical medical emergencies with profound implications for long-term functional outcomes. Identifying reliable prognostic biomarkers has become a key research priority to optimize rehabilitation strategies. Although blood-based nutritional biomarkers are well-established indicators of metabolic status, their specific utility in predicting functional recovery during neurorehabilitation remains insufficiently characterized. In particular, within the specific population of patients with CNS injury, the dynamic changes of these markers and the strength of their independent association with neurological function have not been fully elucidated after accounting for key confounders such as injury type and severity. This retrospective study investigated the correlation between nutrition-related blood biomarkers and functional prognosis in patients with CNS injuries. The study identified albumin, TC, and LDL-C as independent factors associated with the functional prognosis of CNS injury patients, where albumin is a protective factor, TC and LDL-C as risk factors. These results enhance the understanding of the nutritional pathophysiology underlying neurorecovery and provide clinically actionable prognostic insights. Moreover, establishing the specific cutoff values for these biomarkers may give clinicians clearer guidance, which could improve patient outcomes and elevating the overall quality of care for those recovering from CNS injury.

Previous studies have thoroughly investigated the relationship between serum albumin levels and the incidence and progression of stroke. A significant association between higher serum albumin concentrations and improved outcomes in patients with acute stroke was demonstrated by Babu et al. [18]. A 24-year follow-up study further demonstrated that hypoalbuminemia was significantly connected to an elevated risk of total and ischemic strokes [19]. The persistence of albumin’s prognostic value beyond the acute phase suggests sustained pathophysiological relevance. Hypoalbuminemia has been indicated to predict poor prognosis in stroke patients. Moreover, hypoalbuminemia is common during the acute phase of SCI due to severe trauma, surgical interventions, and high infection rates [20]. A previous study on patients with SCI demonstrated that individuals with higher serum albumin levels, measured between 24 h and 1 month after injury, exhibit greater odds of achieving significant neurological recovery [21]. Consistent with these results, this findings confirm that hypoalbuminemia is also correlated with poor functional prognosis in a broader cohort of patients with CNS injury. The robustness of this correlation persisted across subgroup analyses stratified by gender and injury type. Potential mechanisms may involve albumin’s capacity to modulate cerebral hemodynamics and exert direct neuroprotective effects on neurons and glial cells [22]. Furthermore, stroke or severe trauma can trigger a systemic inflammatory response. Activated immune cells, such as macrophages, promote excessive cytokine production during inflammation, which shifts hepatic protein synthesis from albumin toward acute-phase reactants. Therefore, serum albumin serves as a sensitive biomarker reflecting the degree of systemic inflammation [23], associated with the complex prognosis of CNS injury. This suggests that future studies should further elucidate the specific pathways through which albumin influences outcomes—such as by modulating neuroinflammation or serving as a proxy for overall nutritional status—and explore its combined use with more specific neural injury markers, thereby constructing a more comprehensive pathophysiological framework and improved prognostic prediction models.

Previous studies have established correlations between abnormal blood lipid/lipoprotein levels and CNS disease pathogenesis [24, 25]. A meta-analysis including 61 prospective studies and 55,000 cases of vascular deaths reported a positive association between TC and mortality due to ischemic heart disease [26]. While another meta-analysis of 4,512 SCI subjects and 1,252 controls demonstrated significantly higher TC/HDL-C ratio and lower HDL-C levels in patients with SCI, constituting risk factors for cardiovascular comorbidities [27]. LDL-C, in particular, has been a focus in CNS injury research; a globally multicenter study indicated that intensive LDL-C reduction improved cardiovascular prognosis following stroke [28]. Concurrently, a prospective study identified that the interplay of lipids and lipoproteins (TC/HDL-C ratio and TG/HDL-C ratio) was connected to a heightened risk of myocardial infarction and stroke in participants with SCI [29], further reinforcing the link between lipid dysregulation and poor CNS injury prognosis. These studies collectively reinforce the association between TC, LDL-C, and the prognosis of CNS injury. From a mechanistic perspective, cholesterol serves as a fundamental component of cell membranes and is crucial for maintaining membrane fluidity and permeability [30]. This fundamental role of cholesterol indicates that disruptions in cholesterol homeostasis can have cascading effects on cellular processes within the CNS. Cholesterol-driven inflammation appears to influence neuroplasticity by changing membrane properties, vesicular transport, monoamine release, and neuroendocrine functions [31, 32]. Such alterations may impede the recovery process following CNS injury. Additionally, emerging evidence indicates that circulating levels of LDL-C or HDL-C may affect CNS differentiation and function [33]. Given that the CNS is highly dependent on lipid homeostasis for both its structure and function, variations in LDL-C and HDL-C levels could have significant implications for neural repair and rehabilitation. Consequently, TC and LDL-C levels may assist in predicting functional outcomes following CNS injury, with elevated TC and LDL-C being associated with poorer functional outcomes. Notably, while the strength of these associations remained consistent across injury type subgroups, however, total cholesterol showed a pronounced and significant adverse association specifically in hemorrhagic stroke, highlighting a potential injury-specific pathophysiological interaction. Meanwhile a modifying effect of gender was observed, with the associations being significant primarily in male patients. This interaction suggests that sex-specific factors may influence the prognostic utility of these lipid biomarkers, warranting further investigation to optimize their application across diverse patient populations.

Multivariate logistic regression demonstrated that albumin, TC, and LDL-C were closely associated with patient prognosis. Specifically, patients with poor outcomes displayed significantly lower albumin and higher TC and LDL-C levels, indicating that these biomarkers may act as risk factors for adverse prognosis. Optimal cutoff values for albumin, TC, and LDL-C were determined using ROC analysis, aiming to further improve the clinical significance of the results. When patients were categorized based on the derived cutoff values, significant differences in prognosis were observed for albumin, TC, and LDL-C. This further validated the rationality of employing albumin, TC, and LDL-C as prognostic indicators. Previous reports have indicated that lower TC is correlated with a higher risk of stroke [34]; meanwhile, past research has drawn a connection between lower LDL-C and increased stroke risk [28]. This study not only confirmed this established connection but also introduced a new perspective by determining relatively lower cutoff values for TC and LDL-C. This study underscores the remarkable sensitivity of albumin, TC, and LDL-C levels to the physiological disruptions caused by CNS injury. These biomarkers appear to be finely attuned to the body’s response to injury, making them potentially valuable indicators of patient prognosis. Consequently, the cutoff values obtained in this study may serve as useful references for risk statiffication. With multi-center validation, they could potentially aid in identifying high-risk patients and inform personalized rehabilitation strategies and improving long-term care quality.

### Strengths and limitations

A major advantage of this study lies in its inclusion of diverse CNS injury types (SCI and multiple brain injury types), which offers a comprehensive pathological spectrum for assessing prognostic factors and improves the generalizability of this findings. Furthermore, to address methodological limitations commonly found in rehabilitation research, this study utilized the mRFG as a refined outcome. This metric provides elevated sensitivity to functional alterations by accounting for baseline status [35], thereby decreasing ceiling effects and more accurately capturing recovery efficacy [36]. To ensure the credibility of the results, a multifaceted statistical strategy was implemented: Multivariate logistic regression models were undertaken through meticulous adjustment for potential factors, and initial cutoff values were then established. Smooth curve fitting was implemented to clarify variable correlations by eliminating interaction effects. Subgroup and sensitivity analyses further confirmed the robustness and reliability of the study’s conclusions. This comprehensive analytical framework improves the validity of the conclusions and highlights the utility of integrating advanced statistical methods in rehabilitation research.

Several limitations of the study warrant consideration. First, this was a single-center retrospective study, with analyses restricted to routinely available clinical parameters. Although clear inclusion and exclusion criteria were applied, selection bias caused by inherent incomplete data and missing information in retrospective research remained inevitable. Meanwhile, this study did not implement strict homogenization control over the detailed rehabilitation intervention protocols and nutritional support regimens of enrolled patients. Differences in rehabilitation intervention intensity, duration, and regimens, as well as nutritional support strategies, may have confounding effects on patients’ functional prognosis, which cannot be completely eliminated by the retrospective study design. Therefore, the stability and generalizability of these findings need to be further verified in multi-center, large-sample prospective cohorts. Second, although the mRFG offers higher sensitivity to functional changes than the MBI, it does not account for rehabilitation gains related to hospitalization duration, which is a methodological limitation of this study. Future studies should incorporate hospitalization timelines to better capture patients’ rehabilitation progress. Third, only admission blood markers were analyzed, serial blood sampling during rehabilitation is recommended for future studies to monitor dynamic alterations in biomarkers and explore their potential causal relationship with long-term rehabilitation outcomes. Fourth, ROC analyses identified the optimal cutoff values for albumin, TC, and LDL-C for mRFG prediction, with modest AUC values. This suggests that these nutrition-related biomarkers offer only limited predictive performance; therefore, caution is warranted when using them individually for prognostic assessment or as standalone tools. Finally, the findings from the disease subgroup analyses are supportive but not conclusive, underscoring the need for large-scale validation within dedicated, disease-specific cohorts. Future research could explore integrating these blood-based nutritional indicators with other key clinical and imaging parameters, and relying on standardized rehabilitation and nutritional intervention protocols, to assist clinicians in early identification of nutrition-related risks in patients with CNS injury, thereby optimizing individualized intervention strategies and improving patients’ functional prognosis and quality of life.

## Conclusion

This study demonstrates that serum albumin, TC, and LDL-C levels possess independent predictive value for functional prognosis in patients with CNS injury. These biomarkers can be integrated into routine rehabilitation practice through admission blood tests. Albumin, TC, and LDL-C levels enable early risk stratification: patients with low albumin warrant prompt nutritional support, while those with elevated TC or LDL-C may benefit from closer monitoring during rehabilitation. These results emphasize the clinical relevance of monitoring these hematological parameters, which may facilitate early identification and intervention for malnutrition in CNS injury survivors, ultimately enhancing rehabilitation efficacy and quality of life. 

## Supplementary Information

Supplementary Material 1: Table S1. Baseline admission characteristics and univariate analysis of factors associated with prognosis. Table S2. Blood biomarkers at admission and univariate analysis to determine the factors associated with prognosis. Table S3. Multivariate logistic regression of mRFG. Table S4. Bootstrap for variables in the multivariate logistic regression of mRFG. Table S5. Multivariate logistic regression analyses with TC or LDL-C in separate models. Table S6. Validation of prognostic factors by univariate and multivariate linear regression analyses. Table S7. Logistic regression analysis using complete cases (listwise deletion). Fig S1. Testing for nonlinearity of albumin, TC, LDL-C, and mRFG in CNS injury prognosis. Fig S2. Forest plots for the association of albumin, TC, LDL-C, and prognosis.

## Data Availability

The datasets analyzed during the current study are available from the corresponding author upon reasonable request.

## Abbreviations

CNS: central nervous system
SCI: spinal cord injury
ADL: activities of daily living
WHO: World Health Organization
LOS: length of stay
HIE: hypoxic–ischemic encephalopathy
TBI: traumatic brain injury
CHD: coronary heart disease
BMI: body mass index
TLRF: time from lesion to rehabilitation facility
MBI: modified Barthel index
mRFG: the mean relative functional gain
WBC: white blood cell
TC: total cholesterol
TG: triglyceride
HDL: high-density lipoprotein
LDL-C: low-density lipoprotein cholesterol
RCS: restricted cubic spline
ROC: receiver operating characteristic
AUC: area under the curve
OR: odds ratio
CI: confidence interval
