# Supplementary material for: Blood nutrition-related biomarkers for central nervous system injury rehabilitation prognosis: a retrospective study
Source: Lipids Health Dis. 2026 Apr 28;25:150. doi: 10.1186/s12944-026-02957-8 (PMC13267521; doi:10.1186/s12944-026-02957-8)
Supplement: Supplementary file 1 — Supplementary Material 1: Table S1. Baseline admission characteristics and univariate analysis of factors associated with prognosis. Table S2. Blood biomarkers at admission and univariate analysis to determine the factors associated with prognosis. Table S3. Multivariate logistic regression of mRFG. Table S4. Bootstrap for variables in the multivariate logistic regression of mRFG. Table S5. Multivariate logistic regression analyses with TC or LDL-C in separate models. Table S6. Validation of prognostic factors by univariate and multivariate linear regression analyses. Table S7. Logistic regression analysis using complete cases (listwise deletion). Fig S1. Testing for nonlinearity of albumin, TC, LDL-C, and mRFG in CNS injury prognosis. Fig S2. Forest plots for the association of albumin, TC, LDL-C, and prognosis. [file 12944_2026_2957_MOESM1_ESM.zip › Supplementary Files2026.03.10/New-Supplementary Table 3.docx]

**Supplementary Table 3.** Multivariate logistic regression of mRFG.

| **Variables** | **Crude** | | **Model 1** | | **Model 2** | |
| --- | --- | --- | --- | --- | --- | --- |
|  | **OR (95% CI)** | ***P* value** | **OR (95% CI)** | ***P* value** | **OR (95% CI)** | ***P* value** |
| TLRF | 1.001 (1.000~1.002) | 0.054 | 1.001 (1.000~1.002) | **0.041** | 1.001 (1.000~1.002) | **0.038** |
| Injury type |  |  |  |  |  |  |
| SCI | 1 (Ref) |  | 1 (Ref) |  | 1 (Ref) |  |
| Hemorrhagic stroke | 0.634 (0.377~1.066) | 0.086 | 0.553 (0.32~0.956) | **0.034** | 0.498 (0.238~1.045) | 0.065 |
| Ischemic stroke | 0.463 (0.282~0.758) | **0.002** | 0.355 (0.205~0.613) | **<0.001** | 0.313 (0.154~0.637) | **0.001** |
| Other brain injury | 0.526 (0.255~1.086) | **0.082** | 0.586 (0.280~1.226) | 0.156 | 0.462 (0.204~1.046) | 0.064 |
| Smoking |  |  |  |  |  |  |
| No | 1 (Ref) |  | 1 (Ref) |  | 1 (Ref) |  |
| Yes | 0.524 (0.339~0.811) | **0.004** | 0.467 (0.292~0.749) | **0.002** | 0.469 (0.291~0.757) | **0.002** |
| Hemoglobin | 0.986 (0.975~0.997) | **0.014** | 0.986 (0.973~0.998) | **0.020** | 0.988 (0.976~1.001) | 0.067 |
| Total protein | 0.980 (0.947~1.014) | 0.241 | 0.985 (0.951~1.021) | 0.405 | 0.992 (0.957~1.027) | 0.637 |
| Albumin | 0.901 (0.855~0.949) | **<0.001** | 0.905 (0.856~0.957) | **<0.001** | 0.913 (0.862~0.967) | **0.002** |
| Creatinine | 0.997 (0.992~1.003) | 0.385 | 0.997 (0.991~1.003) | 0.344 | 0.998 (0.992~1.005) | 0.613 |
| TC | 1.685 (1.337~2.123) | **<0.001** | 1.74 (1.368~2.214) | **<0.001** | 1.794 (1.389~2.317) | **<0.001** |
| TG | 1.054 (0.903~1.230) | 0.508 | 1.063 (0.895~1.261) | **0.487** | 1.050 (0.904~1.221) | 0.521 |
| LDL-C | 1.618 (1.245~2.102) | **<0.001** | 1.662 (1.267~2.180) | **<0.001** | 1.599 (1.215~2.105) | **<0.001** |

**Notes**: Crude model without any adjustments; Model 1 adjusted for gender, age, education, BMI; Model 2 adjusted for all other factors. Significant values are in bold.

**Abbreviations**: CI, confidence interval; TLRF, time from lesion to the current rehabilitation facility; SCI, spinal cord injury; CHD, coronary heart disease; TC, total cholesterol; TG, triglyceride; LDL-C, low density lipoprotein cholesterol.
