# Supplementary material for: Blood nutrition-related biomarkers for central nervous system injury rehabilitation prognosis: a retrospective study
Source: Lipids Health Dis. 2026 Apr 28;25:150. doi: 10.1186/s12944-026-02957-8 (PMC13267521; doi:10.1186/s12944-026-02957-8)
Supplement: Supplementary file 1 — Supplementary Material 1: Table S1. Baseline admission characteristics and univariate analysis of factors associated with prognosis. Table S2. Blood biomarkers at admission and univariate analysis to determine the factors associated with prognosis. Table S3. Multivariate logistic regression of mRFG. Table S4. Bootstrap for variables in the multivariate logistic regression of mRFG. Table S5. Multivariate logistic regression analyses with TC or LDL-C in separate models. Table S6. Validation of prognostic factors by univariate and multivariate linear regression analyses. Table S7. Logistic regression analysis using complete cases (listwise deletion). Fig S1. Testing for nonlinearity of albumin, TC, LDL-C, and mRFG in CNS injury prognosis. Fig S2. Forest plots for the association of albumin, TC, LDL-C, and prognosis. [file 12944_2026_2957_MOESM1_ESM.zip › Supplementary Files2026.03.10/New-Supplementary Table 7.docx]

**Supplementary Table 7.** Logistic Regression Analysis Using Complete Cases (Listwise Deletion). n=621

| **Variables** | **Univariate** | | | **Multivariate** | | | |
| --- | --- | --- | --- | --- | --- | --- | --- |
|  | **β** | ***P*** | **OR (95% CI)** |  | **β** | ***P*** | **OR (95% CI)** |
| Age | 0.010 | 0.149 | 1.011 (0.993 ~ 1.025) |  |  |  |  |
| LOS | -0.001 | 0.249 | 0.997 (0.993 ~ 1.002) |  |  |  |  |
| Injury type |  |  |  |  |  |  |  |
| SCI |  |  | 1 (Ref) |  |  |  | 1 (Ref) |
| Hemorrhagic stroke strokestrokestroke strokes | -0.355 | 0.217 | 0.701 (0.400 ~ 1.231) |  | -0.189 | 0.527 | 0.828 (0.461 ~ 1.486) |
| Ischemic stroke | -0.626 | **0.022** | 0.534 (0.312 ~ 0.914) |  | -0.325 | 0.272 | 0.722 (0.404 ~ 1.290) |
| Other brain injury | -0.566 | 0.173 | 0.568 (0.251 ~ 1.282) |  | -0.555 | 0.205 | 0.574 (0.243 ~ 1.355) |
| Feeding type |  |  |  |  |  |  |  |
| Oral |  |  | 1 (Ref) |  |  |  |  |
| Enteral nutrition | -0.091 | 0.707 | 0.913 (0.568 ~ 1.468) |  |  |  |  |
| Parenteral nutrition | -1.014 | 0.410 | 0.363 (0.032 ~ 4.055) |  |  |  |  |
| Education |  |  |  |  |  |  |  |
| Low |  |  | 1 (Ref) |  |  |  |  |
| Medium | 0.235 | 0.437 | 1.265 (0.699 ~ 2.289) |  |  |  |  |
| High | -0.153 | 0.635 | 0.858 (0.456 ~ 1.616) |  |  |  |  |
| Gender |  |  |  |  |  |  |  |
| Male |  |  | 1 (Ref) |  |  |  |  |
| Female | 0.305 | 0.258 | 1.357 (0.800 ~ 2.303) |  |  |  |  |
| Smoking |  |  |  |  |  |  |  |
| No |  |  | 1.00 (Reference) |  |  |  |  |
| Yes | -0.298 | 0.259 | 0.742 (0.443 ~ 1.245) |  |  |  |  |
| Diabetes |  |  |  |  |  |  |  |
| No |  |  | 1 (Ref) |  |  |  |  |
| Yes | -0.215 | 0.501 | 0.807 (0.432 ~ 1.507) |  |  |  |  |
| Hypertension |  |  |  |  |  |  |  |
| No |  |  | 1 (Ref) |  |  |  |  |
| Yes | -0.169 | 0.447 | 0.845 (0.546 ~ 1.306) |  |  |  |  |
| CHD |  |  |  |  |  |  |  |
| No |  |  | 1 (Ref) |  |  |  |  |
| Yes | -0.491 | 0.194 | 0.612 (0.292 ~ 1.283) |  |  |  |  |
| Pressure |  |  |  |  |  |  |  |
| No |  |  | 1 (Ref) |  |  |  |  |
| Yes | 0.921 | 0.215 | 2.511 (0.586 ~ 10.756) |  |  |  |  |
| BMI |  |  |  |  |  |  |  |
| Underweight |  |  | 1 (Ref) |  |  |  |  |
| Normal | 0.731 | 0.113 | 2.076 (0.841 ~ 5.126) |  |  |  |  |
| Overweight | 0.476 | 0.324 | 1.610 (0.625 ~ 4.150) |  |  |  |  |
| Obese | 0.965 | 0.268 | 2.625 (0.476 ~ 14.486) |  |  |  |  |
| WBC | -0.004 | 0.867 | 0.996 (0.947 ~ 1.047) |  |  |  |  |
| Hemoglobin | -0.012 | 0.050 | 0.988 (0.975 ~ 1.000) |  |  |  |  |
| Total protein | -0.035 | 0.075 | 0.965 (0.928 ~ 1.004) |  |  |  |  |
| Albumin | -0.092 | **0.002** | 0.912 (0.861 ~ 0.965) |  | -0.109 | **<0.001** | 0.897 (0.844 ~ 0.953) |
| Creatinine | -0.001 | 0.664 | 0.999 (0.992 ~ 1.005) |  |  |  |  |
| HDL | -0.024 | 0.958 | 0.976 (0.396 ~ 2.404) |  |  |  |  |
| LDL-C | 0.488 | **0.001** | 1.630 (1.218 ~ 2.182) |  | 0.884 | **0.041** | 1.413 (1.177 ~ 1.966) |
| TC | 0.547 | **<0.001** | 1.728 (1.333 ~ 2.239) |  | 1.278 | **<0.001** | 3.590 (1.709 ~ 7.541) |
| TG | 0.032 | 0.595 | 1.033 (0.917 ~ 1.163) |  | |  |  |

**Notes:** Data missingness was observed in the following variables: education level (n=1), smoking status (n=2), diabetes (n=10), hypertension (n=21), coronary heart disease (CHD, n=18), intracranial pressure (n=1), and feeding type (n=8). After integrating the dataset, a total of 48 cases with any missing data were excluded from the primary complete-case analysis.

**Abbreviations:** LOS, length of stay; SCI, spinal cord injury; CHD, coronary heart disease; BMI, body mass index; WBC, white blood cell; HDL, high-density lipoprotein; LDL-C, low density lipoprotein cholesterol; TC, total cholesterol; TG, triglyceride.
